# Bacterial Metabolite Reuterin Attenuated LPS-Induced Oxidative Stress and Inflammation Response in HD11 Macrophages

**DOI:** 10.3390/antiox11091662

**Published:** 2022-08-26

**Authors:** Yibin Xu, Xiaoqing Ding, Yuanyuan Wang, Danlei Li, Lingyu Xie, Shuang Liang, Yunfeng Zhang, Weifen Li, Aikun Fu, Xiuan Zhan

**Affiliations:** Key Laboratory of Animal Nutrition and Feed in East China, Ministry of Agriculture, Key Laboratory of Animal Feed and Nutrition of Zhejiang Province, College of Animal Science, Zhejiang University (Zijingang Campus), Hangzhou 310058, China

**Keywords:** reuterin, HD11 cell line, lipopolysaccharide (LPS), inflammation response, oxidative stress

## Abstract

Reuterin is well-known for its broad-spectrum antimicrobial ability, while the other potential bioactivity is not yet clear. The present study aims to investigate the immunomodulatory activity of reuterin on chicken macrophage HD11 cells for the first time and evaluate whether reuterin is able to regulate the lipopolysaccharide-stimulated inflammatory response. The results showed that the safe medication range of reuterin was less than 250 μM. Reuterin treatment for 6 h decreased the transcriptional of *CD86*, *IL-1β* and *iNOS* and increased the expression of *CD206* in a dose-dependent way, but reuterin treatment for 12 h contrary increased the expression of *IL-1β*, *IL-6* and *IL-10*. However, it was noticed that reuterin treatment for 12 h significantly decreased the production of reactive oxygen species (ROS) and suppressed the phagocytosis activity of HD11 macrophages against bacteria. Further, the results showed that preincubation or coincubation with reuterin significantly attenuated the promotive effects of lipopolysaccharide (LPS) on transcription of proinflammatory cytokines (including *IL-1β*, *IL-6* and *TNF-α*) and obviously inhibited nitric oxide (NO) production as well as the protein expression of inducible nitric oxide synthase (*iNOS*). Meanwhile, Mechanism studies implied that reuterin might exert an anti-inflammatory effect on LPS-stimulated cells by downregulating the expression of *TLR4*/*MyD88*/*TRAF6* and blocking the activation of NF-κB as well as MAPKs signaling pathways. Additionally, it was found that both pretreatment and cotreatment with reuterin remarkably inhibited the oxidative stress induced by LPS stimulation by activating the Nrf2/HO-1 signaling pathway and enhancing the activities of antioxidative enzymes. These findings suggested the immunoregulatory function of reuterin and indicated this bacterial metabolite was able to inhibit the inflammation and oxidative stress of HD11 macrophages once exposed to LPS stimulation.

## 1. Introduction

Macrophages are believed to play imperative roles in regulating innate and adaptive immune responses to sustain hemostasis and defend against external pathogens infections [1,2]. The environmental signaling cues could direct macrophages into different phenotypes and endow macrophages with special function status, and this process is usually defined as macrophage polarization. There is a common understanding that macrophages could be activated into two distinct subsets, classically activated (M1) macrophages and alternative activated (M2) macrophages, following the TH1/TH2 paradigm [3,4]. M1 macrophages are characterized by an increased expressional level of *CD80* and *CD86* costimulatory molecules and toll-like receptor 4 (*TLR4*). This kind of macrophages are prone to produce proinflammatory cytokines such as tumor necrosis factor (TNF)-α, interleukin (IL)-1β and IL-6 and produce nitric oxide (NO) or reactive oxygen intermediates (ROI) to protect against pathogens [5,6]. In contrast, M2 polarized macrophages express high levels of *CD204*, *CD163* and *CD206* surface markers. The M2-type macrophages contribute to the production of anti-inflammatory cytokines such as IL-10, transforming growth factor β (TGF-β) and producing polyamines as well as proline to promote inflammation resolution and tissue repair [7]. In vitro macrophages M1 polarization is mainly induced by IFN-γ, colony-stimulating factor (GM-CSF) or lipopolysaccharides from Gram-negative bacteria, while M2 polarization is regulated by IL-4, IL-10 or IL-13 cytokines [8,9].

There are increased researches indicating the crosstalk between probiotics and macrophage polarization. Previous studies indicated that *Lactobacillus johnsonii* NBRC 138952 strain increased the expression of M1-like macrophage marker and enhanced phagocytic activity of Raw264.7 towards *Aggregatibacter actinomycetemcomitans* [10]. Coculture assays of *Lactobacillus rhamnosus* GG (LGG) with bone-marrow-derived macrophages triggered M1 macrophages polarization by activating TLR2/MyD88/MAPK signaling pathway [11]. Meanwhile, further studies unveiled that the immunoregulatory effects of probiotics could be the consequence of specific components from cellular components and metabolites. For example, the exopolysaccharides extracted from *Lactobacillus paracasei* and *Lactobacillus plantarum* effectively directed Raw264.7 macrophages towards M1-type polarization [12]. Cell-free supernatants from different *Lactobacillus* species modulated the cytokine production of human macrophages to varying degrees [13]. Particularly, among the broad microbiota-generated metabolites, butyrate was demonstrated to facilitate the M2 macrophage polarization through activating STAT6 whether in vivo or in vitro [14]. Additionally, recent research demonstrated that extracellular vesicles from *Lactobacillus plantarum* were able to promote differentiation of human THP-1 cells toward M2-like macrophages and attenuated the inflammatory process when exposed to LPS stimulation [15].

Reuterin is a broad antimicrobial agent produced by certain *Lactobacillus reuteri* strains during glycerol metabolism [16]. With the assistance of glycerol dehydratase (GDH), those strains convert glycerol into 3-hydroxypropionaldehyde (3-HPA) [17]. In an aqueous solution, 3-HPA spontaneously transforms into its hydrated or dimer forms. These components keep a dynamic balance, which is therefore concluded as reuterin. Numerous studies show reuterin has an obvious antagonistic effect against a wide of Gram-positive and Gram-negative bacteria [18,19]. One of the proposed antimicrobial mechanisms is that reuterin enable to increase intracellular reactive oxygen species (ROS), which in turn result in oxidative injuries in microorganism [20]. The recent research of Hannah N. Bell [21] exhibited that reuterin could also alter redox balance in colorectal cancer cells and ultimately induce cellular death, which indicated reuterin also affected the activities of eukaryotic cells. Although the high reactivity of ROS with various cellular constituents such as proteins, DNA and lipids could cause cellular damage, it is an important signal mediator toward macrophage activation [22]. Considering the relationship between ROS and macrophage polarization, the hypothesis that reuterin could regulate macrophage polarization by altering the level of ROS is proposed in this article.

Inflammation caused by bacterial infections is a long-term challenge in chicken farming. Although the use of antibiotics has been alleviating the risk of bacterial infection, it also has aggravated bacterial resistance, which was a hidden danger to the safety and health of poultry and even human beings [23,24]. Much research has demonstrated that plenty of natural agents had anti-inflammatory effects on bacterial infections and other beneficial effects [25,26,27], whereas little information was known about reuterin. Therefore, this study aimed to evaluate the immunoregulatory effect of reuterin by detecting polarization-related gene expression, phagocytosis ability and ROS generation based on the chicken macrophages cell line, HD11. Moreover, we further investigated the effect of reuterin treatment (preincubation or cotreatment) on LPS-stimulated HD11 macrophages and detected the potential mechanisms associated with reuterin action so as to promote its application in husbandry production in the future.

## 2. Materials and Methods

### 2.1. Reagents and Chemicals

The standard samples of reuterin were purchased from Aikon Biopharmaceutical R&D Co., Ltd. (Cat. No. AK0038I1, Nanjing, China). Lipopolysaccharides (LPS) from *E. coli* O55:B5 were purchased from Sigma-Aldrich (Cat. No. L2880, St. Louis, MO, USA). The ROS detection probe 2′,7′-Dichlorodihydrofluorescein diacetate (DCFH-DA) was purchased from Aladdin Biochemical technology co. Ltd. (Cat. No. H131224, Shanghai, China).

### 2.2. Cell Culture

Chicken macrophage-like cell line HD11 (kindly provided by Professor Tuoyu Geng, Yangzhou University) was cultured in RPMI 1640 (Roswell Park Memorial Institute 1640) with 10% fetal bovine serum FBS (Gibco, Invitrogen, Carlsbad, CA, USA) and 1% 100 × penicillin-streptomycin (Solarbio, Beijing, China) at 37 °C in a humidified atmosphere of 5% CO_2_. For all experiments, cells were subcultured and passaged once they reached 80–90% confluence.

### 2.3. Cell Viability Assay

The cellular viability was determined by cell counting kit-8 assay (CCK-8, Cat. No. C0038, Beyotime, Shanghai, China). Briefly, cells were seeded into a 96-well plate at the density of 10^4^ cells per well and incubated overnight. Then cells were treated with 200 μL twofold serial dilutions of reuterin-containing completed medium for 12 h. After that, the reuterin-containing medium was discarded, and CCK-8 working solutions were pipetted into a plate for 2 h incubation. The absorbance was measured using a microplate reader (SpectraMax, Molecular Device Co., Sunnyvale, CA, USA) at 450 nm, and cellular viability was calculated by the following method:

$$(\frac{{\mathrm{OD}}_{\mathrm{treatment}}\;{-\;\mathrm{OD}}_{\mathrm{blank}}}{{\mathrm{OD}}_{\mathrm{control}}\;{-\;\mathrm{OD}}_{\mathrm{blank}}})\times 100\%$$

### 2.4. Phagocytosis Assays

The phagocytosis activity was determined by flow cytometry and plate enumeration methods. The overnight activated *E. coli* O157:H7 cultures were centrifuged and washed three times with sterile PBS. The bacterial pellet was harvested and resuspended in 200 μg/mL FITC-PBS solution followed by 1 h incubation. After being stained with FITC, *E. coli* cells were washed three times again with PBS to adjust bacterial concentration at 10^8^ CFU/mL. The HD11 cells were seeded into two 12-well plates at 3~4× 10^5^ cells per well and allowed to grow overnight. After 12 h reuterin pretreatment, supernatants were discarded, and cells were infected with FITC-*E. coli* at 100 multiplicity of infection (MOI) for 40 min in one plate. Then, planktonic bacteria in this plate were discarded, and the infected HD11 cells were washed with sterile PBS three times, followed by 100 μg/mL gentamycin incubation for 1 h to clean the bacteria attached to the surface. After that, cells were harvested to detect phagocytotic bacteria via flow cytometry (BD Biosciences, San Jose, CA, USA). Meanwhile, cells in the other 12-well plate were also infected with unlabeled bacteria at 100 multiplicity of infection (MOI) for 40 min. After washing and antibiotic treatment, cells were lysed with 0.1%-tritonX-100 to enumerate bacterial colonies by the spread plate method.

### 2.5. LPS-Stimulated Inflammatory Model

The cells (3~4 × 10^5^) were plated in a 12-well plate and cultured overnight. After that, cells were exposed to different treatments: incubation with a normal medium (considered as control group); stimulation with 2 μg/mL LPS for 12 h (LPS group); preincubation with 250 μM reuterin for 12 h followed by 2 μg/mL LPS stimulation for another 12 h (pre-LPS group); and co-incubation with 2 μg/mL LPS and 250 μM reuterin for 12 h (co-LPS group).

### 2.6. Detection of Cellular ROS

The contents of reactive oxygen species (ROS) were detected by both fluorescence microplate reader and flow cytometry with a DCFH-DA detection probe. In brief, 3~4 × 10^5^ HD11 cells were planted in 12-well plates and incubated overnight. After according treatments, cells were washed with sterile PBS and then incubated with 20 μM DCFH-DA (dissolved in PBS) without light for 40 min at 37 °C. Then, cells were washed three times to remove the abundant dye and harvested to conduct flow cytometry (BD Biosciences, San Jose, CA, USA) and observed the fluorescence morphology by inverted fluorescence microscope (Micro-shot Technology Co., Ltd., Guangzhou, China). Meanwhile, the fluorescence was detected using a microreader with 488 nm excitation/525 nm emission, and the background fluorescence of PBS and autofluorescence of cells without DCFH-DA staining were also measured to calculate the net fluorescence emitted.

### 2.7. Measurement of Nitric Oxide

After different treatments, cell supernatants were collected, and the contents of nitric oxide in the supernatants were measured according to the manufacturer’s instructions for nitric oxide (NO) assay kit (cat# A013-1-1, Jiancheng bioengineering institute, Nanjing, China).

### 2.8. Analysis of Oxidative Stress Indices

The oxidative stress parameters of Malondialdehyde (MDA), total antioxidant capacity (T-AOC), glutathione peroxidase (GSH-Px) and total superoxide dismutase (T-SOD) were measured by microscale MDA assay kit (TBA method, A003-2-2), T-AOC assay kit (ABTS method, A015-2-1), GSH-Px assay kit (Colorimetric method, A005-1-2) and T-SOD assay kit (Hydroxylamine method, A001-1-1), respectively. All assay kits were purchased from Nanjing Jiancheng Bioengineering Institute. After different treatments, the cellular supernatants were discarded, and the cells were washed with sterile PBS three times, followed by 0.1% triton-X100 incubation to lyse cells. The cellular lysis buffers were collected and analyzed according to the manufacturer’s instructions.

### 2.9. Real-Time Quantitative PCR Assay

Total RNA was extracted from HD11 cells using the TRIzol reagent (Cat. No. 9108, Takara Biotechnology, Dalian, China) and then quantified and qualified by NanoDrop 2000 (Thermo Fisher Scientific, Wilmington, DE, USA). After diluted to the same concentration with RNase-free water, 1 μg of total RNA was converted into cDNA via HiScript ΙΙ1st Stand cDNA Synthesis Kit (Cat. No. R212, Vazyme, Nanjing, China). The cDNA samples were amplified utilizing ChamQ SYBR qPCR Master Mix (Cat. No. Q341, Vazyme, Nanjing, China) and monitored at CFX real-time PCR system (Bio-Rad, Foster, CA, USA). The reaction procedures were settled as follows: 95 °C denaturation for 30 s, followed by 35 cycles of 95 °C for 5 s, 60 °C annealing and extension for 30 s, and melt curve to confirm the amplification specificity. The relative expression levels of target genes were calculated by $2^{-\Delta \Delta Ct}$ method. Three independent experiments were performed. All primers are listed in Table 1, and β-actin was regarded as the reference gene.

### 2.10. Immunofluorescence Staining

Cells (3~4 × 10^5^) were planted into a 12-well plate loaded with tissue culture-treated glass coverslips and cultured overnight. After being pretreated with reuterin and LPS stimulation, cells were fixed with 4% paraformaldehyde and permeabilized with 0.5% Triton X-100 and blocked with 5% bovine serum albumin. Then, cells were incubated with the primary NF-κB p65 subunit antibody (Abcam, Cambridge, UK, 1:100 dilution) at 4 °C overnight, subsequently incubated with Cy3-conjugated Goat anti-rabbit secondary antibody (Servicebio Technology Co., Ltd., Wuhan, China) for 1 h at room temperature, and stained with 4,6-diamidino-2 phenylindole (DAPI, Servicebio Technology Co., Ltd., Wuhan, China) for 5 min. The nuclear translocation of NF-κB was detected in the fluorescence intensity of the p65 subunit in nuclear under a microscope (Nikon digital eclipse C1, Tokyo, Japan).

### 2.11. Western Blotting Analysis

Cells were washed with pre-cooling PBS and lysed with RIPA buffer containing a cocktail of protease and phosphatase inhibitors (Beyotime, Shanghai, China). The protein concentrations of cellular lysis were detected with the bicinchoninic acid (BCA) method according to the instructions (Cat. No. A045-4-2, Jiancheng bioengineering institute, Nanjing, China). Then samples were adjusted to the same concentration and denatured with 5× DualColor Protein Loading Buffer (Cat. No. FD002, Fude biological technology Co., Ltd., Hangzhou, China). Protein was separated by 12% SDS-PAGE gel and transferred into a nitrocellulose (NC) filter membrane. Membranes were blocked with 5% skimmed milk for 2 h at room temperature and incubated with primary antibody at 4 °C overnight. After three-time washing with Tris-buffered saline containing Tween 20 (TBST), membranes were incubated with horseradish peroxidase (HRP)-conjugated goat anti-rabbit IgG secondary antibodies (Abclonal, Wuhan, China) for another 2 h at room temperature. The protein blots were visualized by the FDbio-Pico ECL kit (Fude biological technology Co., Ltd., Hangzhou, China), and the β-actin was used as a housekeeping protein to normalize the expression of target proteins. For the protein expressions of MAPKs, the same membrane was firstly probed with antibody against its phosphorylation status and then striped with stripping buffer (purchased from Beyotime Biotechnology, cat#P0025, Shanghai, China) to reprobe with antibody against total form as a loading control. The Western blot membranes were analyzed by ImageJ to measure the band intensity within the same area for each lane. All used primary antibodies in this study are listed in Table 2.

### 2.12. Statistical Analysis

All experiments were performed at least three times. Data were analyzed by SPSS Version 20.0 (SPSS Inc., Chicago, IL, USA) and expressed as the mean ± standard deviation (SD). Differences between groups were assessed using analysis of variance followed by ANOVA-Tukey’s post hoc test, and *p* < 0.05 was considered a statistically significant difference.

## 3. Results

### 3.1. Effect of Reuterin on Cell Viability of HD11 Cell Line

**Figure 1:**
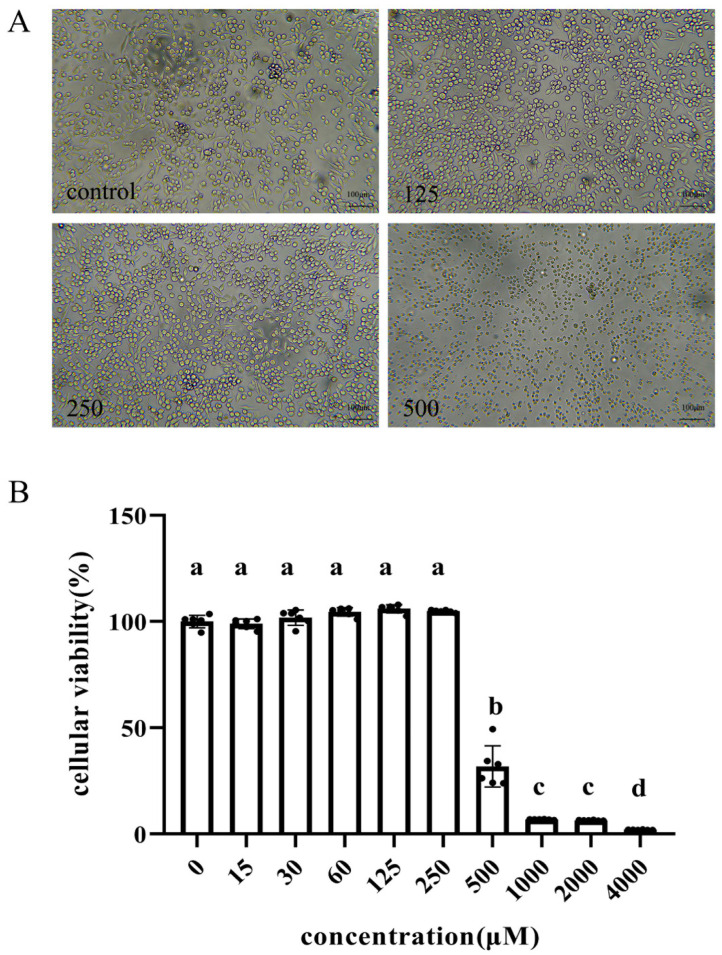
Effects of reuterin on the cellular viability of HD11 cells. Cells were incubated with different concentrations of reuterin (0 to 4000 μM) for 12 h. (**A**) The representative cellular morphological changes were observed in microscope with 100× magnification. Scale bar = 100 μm. (**B**) Cell viability was measured by CCK8 assay. Data are expressed as the mean ± SD with six independent replicates (*n* = 6). Different lowercase letters above column mean significant difference between groups (*p <* 0.05).

The CCK-8 results displayed that compared with the control, the cellular viability of HD11 macrophages is significantly decreased once reuterin concentration is over 250 μM (Figure 1B). In the meanwhile, the microscopy observation exhibited the HD11 cells kept the normal structure and morphology but once exposed to 500 μM for 12 h, HD11 cells were obviously shrunk and cytoplasmic vacuolation, indicating that reuterin above 500 μM is toxic for HD11 cells (Figure 1A).

### 3.2. Effects of Reuterin on the Expressional Levels of Polarization-Associated Genes of HD11 Cells

**Figure 2:**
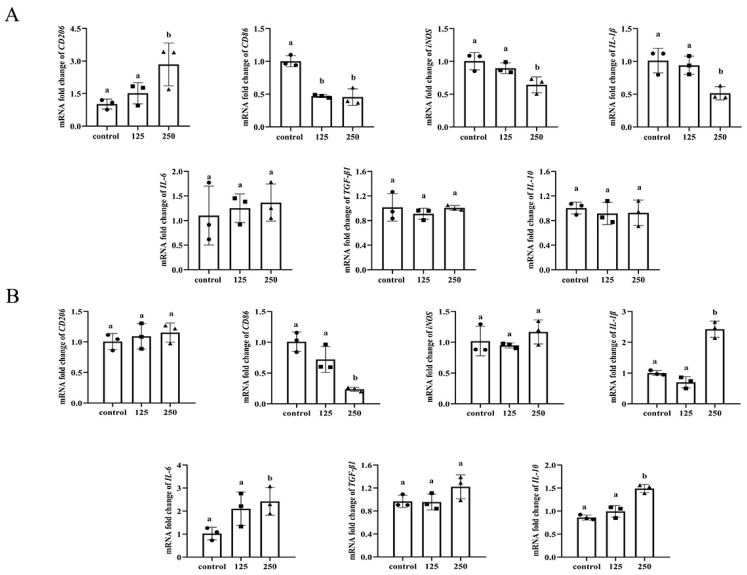
Effects of reuterin on the expression of polarization-associated genes in HD11 cells. Cells were treated with the indicated concentrations of reuterin at different times, and each concentration was independently performed with three replicates (*n* = 3). (**A**) The polarization-associated genes expression of HD11 cells after incubated with reuterin for 6 h. (**B**) The polarization-associated genes expression of HD11 cells after incubated with reuterin for 12 h. Each group contained three biological replicates, and data were shown as mean ± SD and means denoted by a different letter indicate significant differences between treatments (*p* < 0.05).

To assess the effect of reuterin on macrophage polarization, cells were treated with reuterin at 0 (control), 125 and 250 μM for different times (6 h and 12 h) and detected the expression levels of M1- and M2-polarization marker genes. The results showed that 6 h reuterin incubation significantly increased the expression of M2-associated polarization surface marker *CD206* and decreased the expression of M1-associated surface marker *CD86*. Meanwhile, 6 h reuterin incubation also significantly inhibited the expression of internal M1 makers (*iNOS* and *IL-1β*) while having little effect on M2 markers, including *TGF-β1* and *IL-10* (Figure 2A). Additionally, reuterin treatment for 12 h significantly increased the expression of *IL-10* and decreased the expression of *CD86* while unexpectedly upregulating the expression of *IL-1β* and *IL-6* (Figure 2B).

### 3.3. Reuterin Suppressed ROS Production and Phagocytosis Activity of HD11 Cells

**Figure 3:**
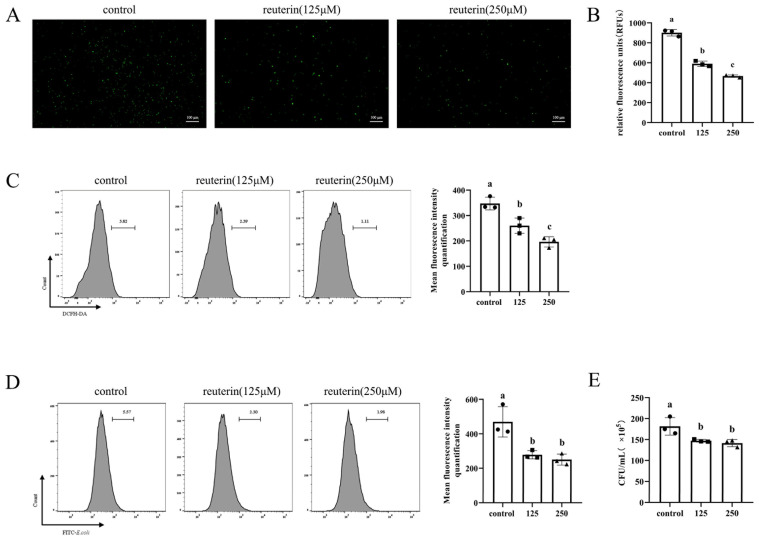
Effects of reuterin on phagocytosis activity in HD11 macrophages. Cells were pretreated with 0, 125 and 250 μM reuterin for 12 h and then infected with FITC-labeled *E. coli* for 40 min at 100 MOI. (**A**) Epifluorescence images of ROS production in HD11 cells after incubated with reuterin for 12 h. HD11 cells were monitored and pictured at 40 × magnification. Scale bar = 100 μm. (**B**) Quantification results of cellular ROS were expressed as the relative fluorescence unit (RFU). Each concentration contained three replicates (*n* = 3). (**C**) Flow cytometry and statistical result of ROS in HD11 cells. Each group contained three tests (*n* = 3). (**D**) Flow cytometry and statistical result of phagocytosis in HD11 cells. Three replicates were conducted in each group of flow cytometry assays (*n* = 3). The mean fluorescence intensity was quantified with FITC-positive population. (**E**) The phagocytic bacterial colonies of HD11 macrophages after *E. coli* infections. The serial ten-fold cellular lysis dilutions were spread on LB agar plate in duplicate and incubated at 37 °C overnight. Counting the colonies from the dilution with plates giving counts between 20 and 200 and taking the average into statistical analysis. Each group contained three biological replicates, and data were shown as mean ± SD and means denoted by a different letter indicate significant differences between treatments (*p* < 0.05).

The fluorochrome DCFH-DA was utilized to detect the production of cellular ROS. As shown in Figure 3A, compared with a normal medium, reuterin incubation for 12 h significantly decreased the positive cell in the field of observation. Meanwhile, the quantification assays of flowcytometry and microplate reader (Figure 3B,C) suggested the incubation with 250 μM reuterin more effectively inhibited the ROS production than 125 μM.

To assess the effect of reuterin on phagocytosis, the *E. coli* O157:H7 cells were collected and labeled with FITC and then incubated with HD11 cells at 100 MOI. The flow cytometry results (Figure 3D) showed that compared with the control group, the FITC-positive population was significantly decreased in reuterin treatment groups, and there was little difference between 125 μM and 250 μM reuterin treatments. Consistently, the result of plate enumeration demonstrated that the incubation with 125 μM and 250 μM reuterin decreased the intracellular *E. coli* cell number from 1.82 × 10^7^ CFU/mL to 1.47 × 10^7^ and 1.42 × 10^7^ CFU/mL (Figure 3E).

### 3.4. Pretreatment and Cotreatment of HD11 Macrophages with Reuterin Inhibited Nitric Oxide Production When Exposed to LPS Stimulation

**Figure 4:**
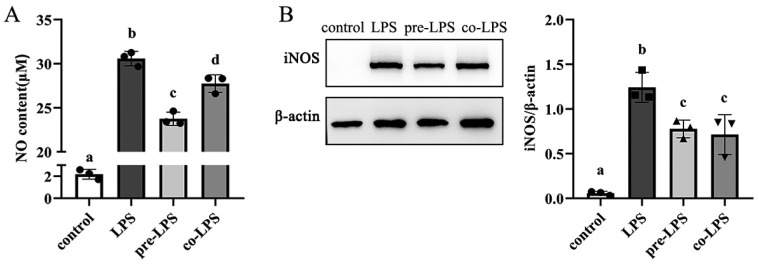
The effect of reuterin on the nitrite production of HD11 cells. The HD11 macrophages were respectively pretreated with 250 μM retuterin for 12 h before LPS stimulation or cotreated with reuterin and LPS for 12 h. Control cells were cultured with normal medium without treatment. (**A**) Concentrations of nitric oxide in cellular supernatants from different groups. (**B**) The protein expression of iNOS of HD11 macrophages in different groups. Experimental results were presented as mean ± SD (*n* = 3 replicates per group); different letters above column indicated the significant difference between groups (*p* < 0.05).

In order to evaluate whether reuterin treatment modulated the inflammatory response in LPS-stimulated HD11 cells, the concentration of inflammatory marker, nitric oxide (NO) in supernatants was detected. As shown in Figure 4A, whether cells were pretreated with 250 μM reuterin or cotreated with 250 μM reuterin at the same time as LPS stimulation, the nitric oxide concentrations were significantly decreased from 33.93 ± 0.25 μM in LPS groups to 28.57 ± 1.02 μM in the pre-LPS group or 30.03 ± 0.42 μM in the co-LPS group. In addition, the Western blot result (Figure 4B) displayed that pretreatment or cotreatment with reuterin obviously inhibited the expression of iNOS compared with the LPS-exposed cells.

### 3.5. Reuterin Inhibited the Oxidative Stress in LPS-Stimulated HD11 Cells by Nrf2/HO-1 Signaling Pathway

**Figure 5:**
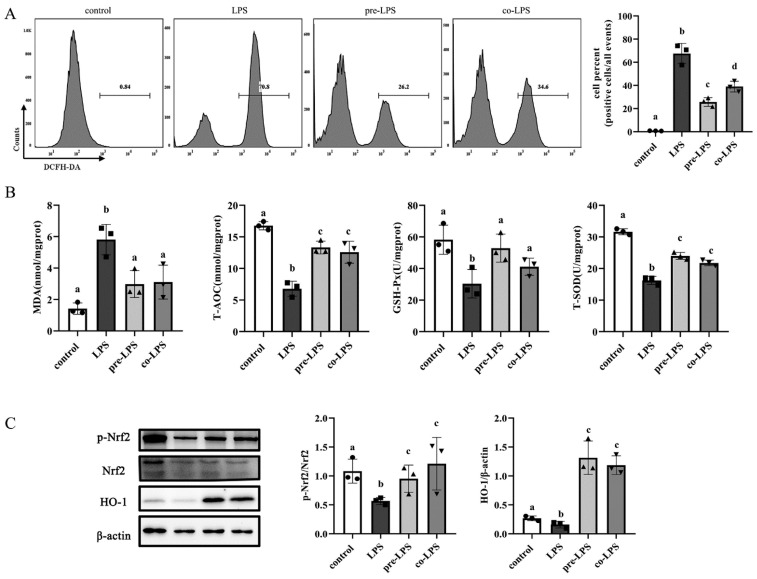
Effects of reuterin on LPS-stimulated oxidative stress in HD11 macrophages. After growing at adequate confluency, the HD11 macrophages were assigned to different treatments: normal medium incubation (control group) 2μg/mL LPS stimulation (LPS group), preincubation with 250 μM reuterin for 12 h and then exposure to LPS stimulation for another 12 h (pre-LPS group), simultaneously treated with 250 μM reuterin and LPS (co-LPS group) for 12 h. Columns marked with different letters indicated the significant difference between groups (*p* < 0.05). (**A**) The flow cytometry results of HD11 cells after being exposed to different treatments. The percentage of ROS-positive cells in each group were expressed as mean ± SD (*n* = 3). (**B**) The effects of reuterin on oxidative stress parameters in LPS-treated HD11 cells. The contents of MDA and activities of T-AOC, GSH-Px and T-SOD in cellular lysis buffer were measured, and data were represented as mean ± SD (*n* = 3). (**C**) The effects of reuterin on the Nrf2/HO-1 signaling pathways. Phosphorylation of p-Nrf2, total Nrf2 and HO-1 were determined by Western blot assay. Quantitative analysis for relative phosphorylation level of Nrf2 and HO-1 was performed by normalizing the total Nrf2 and β-actin, respectively. Experimental results were shown as mean ± SD (*n* = 3).

In Figure 5A, the flowcytometry result showed that LPS (2 μg/mL) treatment markedly increased the intracellular ROS generation while reuterin treatment, especially preincubation with cells, significantly decreased the LPS-induced ROS accumulation. In Figure 5B, reuterin pretreatment and cotreatment significantly decreased the level of MDA, a common oxidative damage marker. Additionally, pretreatment and cotreatment of LPS-induced macrophages with reuterin significantly enhanced the level of T-AOC and increased antioxidant enzyme activities, including GSH-Px and T-SOD, when compared to the model group. Similarly, Western blot assays demonstrated that both pretreatment and cotreatment with reuterin significantly activated the expression of nuclear factor erythroid 2-related factor 2 expression (Nrf2) and increased the expression of downstream heme oxygenase-1 (HO-1) compared to those in LPS-stimulated cells (Figure 5C). Taken together, these assays indicated that reuterin treatment was able to suppress the LPS-induced oxidative stress by activating Nrf2/HO-1 signaling pathway and increasing the activity of antioxidative enzymes.

### 3.6. Reuterin Inhibited the Inflammatory Response in LPS-Stimulated HD11 Cells

**Figure 6:**
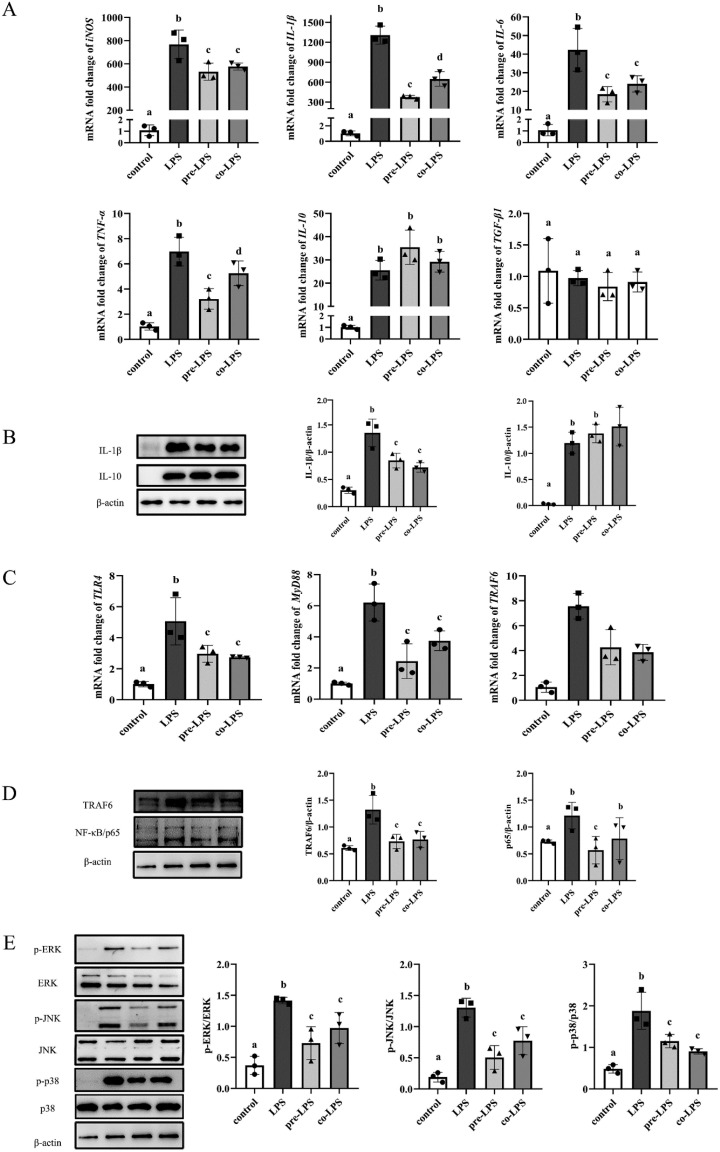
Effects of reuterin on LPS-stimulated inflammation response in HD11 macrophages. HD11 macrophages were cultured in a normal medium (control group) or stimulated with LPS alone for 12 h (LPS group), or pretreated with 250 μM reuterin for 12 h and then exposed to LPS stimulation for another 12 h (pre-LPS), or incubated with LPS and reuterin together for 12 h (co-LPS group). Values were expressed ± SD with three independent replicates (*n* = 3). The significant differences between groups were marked with different lowercase letters (*p* < 0.05). (**A**) The transcriptional levels of inflammation-related genes in different groups. (**B**) Effects of reuterin on the IL-1β and IL-10 protein expression in LPS-stimulated HD11 macrophages. (**C**) Effects of reuterin on the activation of TLR4/MyD88 signaling pathways. The transcription of TLR4, MyD88 and TRAF6 were detected by qRT-PCR. (**D**) Effects of reuterin on the protein expression of TRAF6 and NF-κB. (**E**) Effects of reuterin on the activation of MAPKs signaling pathways. The phosphorylation levels of ERK, JNK and p38 were respectively normalized to total ERK, JNK and p38.

As shown in Figure 6A, LPS exposure not only dramatically enhanced the expression of proinflammation-associated genes expression including *iNOS*, *IL-1β*, *IL-6* and *TNF-α* but also upregulated the expression of anti-inflammatory cytokine *IL-10* compared to that of control group. Meanwhile, reuterin pretreatment and cotreatment significantly inhibited the expression of proinflammation-related genes in LPS-stimulated cells, and preincubation with reuterin has more inhibitory effects on gene expression of *IL-1β* and *TNF-α* when compared with cotreatment. Further, the western blot results declared that LPS stimulation significantly increased the protein expressions of IL-1β and IL-10 compared to the control group. However, the increased IL-1β protein expression was significantly inhibited by both reuterin pretreatment and cotreatment, while the IL-10 protein expression was slightly increased by reuterin treatment (Figure 6B). Since the TLR4/MyD88/NF-κB and MAPKs signaling pathways were always associated with LPS-promoted proinflammatory response, the gene and protein expressions of related molecules were detected in HD11 cells. As shown in Figure 6C, the transcriptional levels of *TLR4*, *MyD88* and *TRAF6* were significantly increased by LPS stimulation, while reuterin treatment restrained LPS-induced increase in these mRNA levels. Meanwhile, pretreatment and cotreatment with reuterin also significantly suppressed the protein expression of TRAF6 and NF-κB in LPS-stimulated HD11 macrophages (Figure 6D). Additionally, the western blot analysis revealed that although ERK, JNK and p38 were activated by exposure of macrophages to LPS (Figure 6E), reuterin pretreatment and cotreatment also significantly curtailed the phosphorylation activation of above MAPKs.

### 3.7. Reuterin Inhibited the LPS-Stimulated NF-κB p65 Nuclear Translocation in HD11 Cells

**Figure 7:**
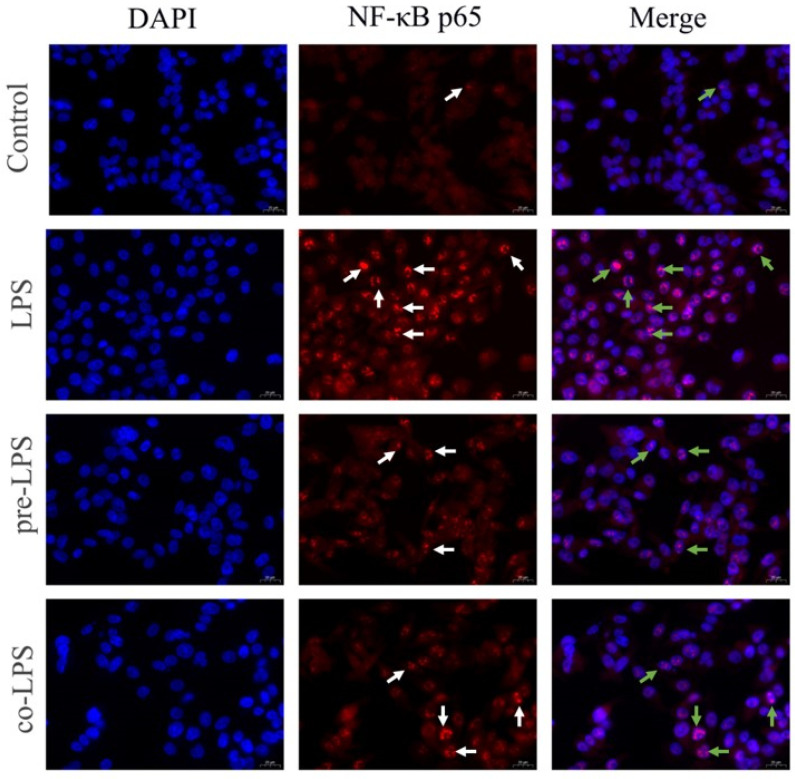
Reuterin inhibited LPS-stimulated NF-κB p65 subunit nuclear translocation in HD11 macrophages. The nuclei were DAPI-stained (blue), and the p65 of treated cells was visualized through immunofluorescence staining with anti-p65(red) antibody. Scale bar = 20 μm. The red area (representative of the area that contained p65) and blue area (representative of the nucleus part that is DAPI conjugated) images were overlaid to create a purple fluorescence in areas of colocalization. White arrows exhibited the p65 immunolabeling in HD11 cells; green arrows indicated the colocalization of p65 immunolabeling within the nucleus.

Next, the location of the p65 protein was detected by an immunofluorescence test. As shown in Figure 7, untreated HD11 cells of NF-κB p65 were mainly distributed in the cytoplasm, while LPS stimulation triggered the abundant translocation of p65 protein into the nucleus. However, the translocation of p65 protein was inhibited by both reuterin pretreatment and cotreatment compared to that of LPS-stimulated HD11 cells.

## 4. Discussion

Chicken is an important farming animal for meeting human dietary nutrition requirements. For a long time, antibiotics have been widely used in poultry production to prevent pathogens infection and accompanying diseases. Meanwhile, companying with the concerns about the presence and delivery of antibiotic-resistance bacteria between animals and humans, searching for other safe alternatives to control bacterial infections is imperative for common health. As mentioned above, macrophages are the first defense line against external pathogens and play a vital role in regulating the process of inflammation. The strategies targeting macrophages’ abilities to enhance host immune function to defend against infections and thus reduce the utilization of antibiotics have attracted increasing attention. Chicken HD11 macrophages are immortalized cell lines [28] and share many similarities with normal chicken macrophages. They have been widely used as a model in vitro in the study of infection and immunobiology to enhance understanding of the underlying molecular mechanisms [29,30].

Probiotics have been defined as a group of microorganisms that are able to benefit host health at an adequate administration amount. Recently, more and more evidence has shown that probiotics are able to directly modulate the functions of macrophages and regulate the host’s innate and adaptive immune response. For example, recent studies performed by Dayong Ren [31] and A. de Moreno de LeBlanc [32] displayed that long-term probiotic administration could increase the phagocytosis ability and M1 polarization of peritoneal macrophages. Additionally, Cao et al. [33] reported that oral *Bacillus amyloliquefaciens* 06 administration could simultaneously enhance the protein expression of M1 and M2 polarization marker, *iNOS*, *Arg* and *CD206* (mannose receptor) in ileum mucosa. Moreover, emerging evidence demonstrated that probiotics could exert an anti-inflammatory effect on maintaining immune homeostasis through regulating the conversion of macrophage polarization when the host is under pathological conditions. For instance, a mixture of probiotic strains *Bifidobacterium longum* CH57 and *Lactobacillus brevis* CH23 have been proved to ameliorate 2, 4, 6-trinitrobenzene sulfonic acid (TNBS)-induced colitis by inhibiting the expression of M1 macrophages marker *IL-1β*, *TNF-α* and increasing the M2 macrophage markers *IL-10*, *Arg-1* and *CD204* [34]. Likewise, a study by Jang [15] declared *Lactobacillus plantarum* CLP-0611 effectively inhibited the TNBS-induced intestinal inflammation and promoted the conversion of M1 macrophages into M2 macrophages.

The immunoregulatory effect of probiotics could be attributed to their specific metabolites to a great extent. Previous research showed that lactic acid bacteria-derived α-linolenic acid metabolites, such as 13-hydroxy-9(Z), 15(Z)-octadecadienoic acid (13-OH) and 13-oxo-9(Z), 15(Z)-octadecadienoic acid (13-oxo) promote the differentiation of M2 macrophages through activation of long-chain fatty acid receptor GPCR40/ERK signaling pathway [35]. Additionally, the short-chain fatty acids (SCFAs) produced by probiotics or other commensal bacteria have been demonstrated to participate in lots of physiological activities and obsess various bioactive functions. In the Raw264.7 cell model, Liu et al. demonstrated that three types of SCFAs, sodium acetate, sodium propionate and sodium butyrate, are capable of inhibiting the production of M1 macrophage-associated cytokines and enhancing the production of M2 macrophage marker *IL-10*. In addition, both sodium acetate and sodium butyrate enable to attenuate LPS-induced proinflammatory response [36], and the inhibition of histone deacetylase and nuclear factor κB (NF-κB) might be the underlying mechanism of SCFAs immunoregulatory activity [37,38]. Meanwhile, the crosstalk between intestinal immune cells and bacteria-derived amino acid metabolites, especially indoles and indole-related derivatives, has also attracted an abundance of notification. Research from Marta Wlodarska et al. displayed that indole-3-propionic acid (IPA) produced by commensal *peptostreptococcus* species obviously enhanced the anti-inflammatory ability of bone marrow-derived macrophages against LPS stimulus [39]. A similar experiment from Smitha Krishnan exhibited that indole-3-acetate inhibited the expression of proinflammatory indicators in macrophages and suppressed the macrophage infiltration to chemokine MCP-1 [40].

*Limosilactobacillus reuteri* is a Gram-positive heterofermentative bacterium widely present in the gastrointestinal tract of vertebrates. Among them, certain strains can produce a bioactive molecule named reuterin. It exhibited obvious bactericidal effects against various pathogens, such as *Escherichia coli*, *Salmonella enteritis*, *Campylobacter jejuni* and *Clostridium perfringens*. Previous research indicated two potential mechanisms involved in the broad-spectrum antibacterial activities of reuterin. Talarico and Dobrogosz [41] suggested that reuterin could interfere with DNA synthesis by inhibiting the ribonucleotide reductase activity. Schaefer [20] and Engevik et al. [42] postulated that reuterin enables the consumption of the thiol-containing antioxidants and disrupts the redox balance in bacteria. Likewise, similar results in various human colorectal cancer cell lines declared trace concentration of reuterin significantly inhibited those cell growth and induced cell death due to elevated ROS [21]. However, to our knowledge, research about the effects of reuterin on macrophage function is still scarce.

Herein, results indicated HD11 macrophage is tolerant to 0~250 μM reuterin, and reuterin at or over 500 μM exhibited a significantly cytotoxic effect, leading to cellular shrinking and death. Although the bactericidal activities of reuterin have been well documented, specific mechanisms involved with reuterin cytotoxicity have yet to be explored and deserve more investigation. The assays of quantitative real-time PCR (qPCR) demonstrated that 6 h reuterin incubation significantly decreased the expression of M1 markers, including *IL-1β* and *iNOS,* through a dose-dependent way, which were increased in HD11 cells exposed to 12 h reuterin treatment. Moreover, reuterin incubation for 12 h also significantly decreased the expression of *CD86* but increased the expression *IL-10*. The mechanisms underlying the dynamic expression changes of polarization-associated genes in reuterin-exposed HD11 macrophages remain for further studies to explain.

Furthermore, although previous indicated that reuterin enable to increase ROS production [21], our studies suggested that reuterin effectively suppressed cellular ROS in HD11 cells in a dose-dependent way. The contrary results might attribute to the different tolerance to reuterin between specific cell lines and biphasic dose responses of reuterin. Phagocytosis is essential for macrophages to engulf and kill the invasive bacteria and is characterized as a feature of macrophage polarization. In our study, the flow cytometry and spread plate results demonstrated that reuterin incubation for 12 h inhibited the ability of HD11 macrophages to swallow *E. coli,* and 250 μM reuterin is more effective than 125 μM. Taken together, it was suggested that reuterin was inclined to induce the M2-like macrophages phenotype of HD11 cells.

Lipopolysaccharide (LPS) is a large molecule from the outer membrane of Gram-negative bacteria. LPS, as a pathogen-associated molecular pattern molecule, induces local or system inflammatory response and is widely utilized in vivo or vitro studies for evaluation of the immunoregulatory activity of bioactive molecules [43]. It is well-known that LPS enables to activate M1 polarization through TLR4 signaling pathways and increase the production of reactive oxygen species and reactive nitrogen species, thus aggravating inflammation. Interestingly, pretreatment or cotreatment with reuterin significantly suppressed the oxidative stress and nitric oxide (NO) production when HD11 cells were stimulated by 2μg/mL LPS. Indeed, the further results indicated that the elevated transcriptional levels of proinflammatory cytokines, including *IL-1β*, *IL-6* and *TNF-α* induced by LPS, were significantly inhibited by reuterin, especially the pretreatment. Interestingly, the expression of *IL-10* in HD11 cells was significantly increased after exposure to LPS stimuli, which is partially consistent with previous findings on mouser Raw264.7 macrophages and human alveolar macrophages [44,45]. Although reuterin treatment also increased the expression of *IL-10*, there is no significant difference compared to that in single LPS-treated cells.

In addition, excess ROS production induced by LPS stimulation between inflammatory responses always triggered oxidative stress. In our study, reuterin exhibited obvious effects on alleviating the oxidative stress induced by LPS exposure in HD11 macrophages. It was shown that pretreatment and cotreatment of LPS-induced HD11 cells with reuterin not only significantly decreased the ROS and MDA production but also enhanced the activity of downstream antioxidant enzymes. Previous research pointed out that transcriptional factor nuclear factor erythroid 2-related factor 2 (Nrf2) not only mediated the oxidative stress response by regulating the expression of antioxidative enzymes but also attenuated the proinflammatory response via inhibiting the transcription of *IL-6* and *IL-1β* [46,47]. The current results revealed that reuterin treatment (pretreatment and cotreatment) was able to promote the activation of Nrf2 and increase the expression downstream of heme oxygenase-1 (HO-1).

In addition, numerous studies have demonstrated that TLR4/MyD88/NF-κB and MAPKs signaling pathways play a critical role in the regulation of macrophages’ inflammatory responses towards pathogens or other stimuli [48]. These mentioned signaling pathways enabled to regulate the expression of a series of inflammation-related genes such as *IL-1β*, *IL-6*, *TNF-α* and *IL-10* and regulate the progress of inflammation. In this study, it was found that pretreatment and cotreatment of reuterin significantly inhibited the LPS-induced transcriptional increase in *TLR4*, *MyD88* and *TRAF6*. Additionally, reuterin treatment also suppressed the protein expression of NF-κB subunit p65 and curtailed its translocation into nuclear in LPS-stimulated cells, which indicated that reuterin might inhibit the LPS-induced activation of TLR4/MyD88/TRAF6/NF-κB signaling pathway. Meanwhile, pretreatment and cotreatment of reuterin also significantly inhibited the LPS-triggered activation of ERK, JNK and p38, suggesting that reuterin also exerted an anti-inflammatory effect through the inhibition of MAPK phosphorylation.

## 5. Conclusions

In summary, we discovered a natural metabolite from *Limosilactobacillus reuteri;* reuterin shows immunoregulatory effects in HD11 cells and tends to induce M2-type polarization, which is mainly characterized by decreased ROS production and phagocytosis activity. Additionally, reuterin exhibits evidently antioxidative and anti-inflammatory effects by inhibition of Nrf2/HO-1, NF-κB, and MAPKs pathways in LPS-exposed cells. Taken together, the current results enlighten the potential of reuterin to alleviate inflammation-related diseases, which also provide a new insight to explain the anti-inflammatory effect of probiotics.

## Figures and Tables

**Table 1:** The primers used in this study.

Gene Name	Accession Number	Forward Sequence (5′-3′)	Reward Sequence (5′-3′)
*TLR4*	NM_001030693	ATCTTTCAAGGTGCCACATC	GGATATGCTTGTTTCCACCA
*MyD88*	NM_001030962	CTGGCATCTTCTGAGTAGT	TTCCTTATAGTTCTGGCTTCT
*TRAF6*	XM_040673311	TCTGTTTGTCCACACGATGC	TATCTCTGGCTTGGCTTCCA
*iNOS*	NM_204961	AAAGATGATGCCAAATTACACA	TCCGACAATTGATAACCTCC
*CD206*	NM_001397660	GAGGACTGCGTTGTTATGA	TCTTCTGTCGGTGCTTCT
*CD86*	NM_001037839	GGATGTCTTACAGGATGCT	CTGCTCTCCAAGGTGAAG
*IL-1β*	NM_204524	ACTGGGCATCAAGGGCTA	GGTAGAAGATGAAGCGGGTC
*IL-6*	NM_204628	ATCCTCTGTTACCAATCTGC	ACATTTTCTTTGGCGTTGAC
*IL-10*	NM_001004414	TGCTGCGCTTCTACACAGAT	TGGCTTTGCTCCTCTTCTCG
*TGF-β1*	NM_001318456	GATGGACCCGATGAGTATTG	CGTTGAACACGAAGAAGATG
*β-actin*	NM_205518	GAGAAATTGTGCGTGACATCA	CCTGAACCTCTCATTGCCA

**Table 2:** The antibody information used in the present study.

Antibody Name	Dilution Ratio	Manufacturers
iNOS	1:1000	HuaAn Biotechnology Co., Ltd., Hangzhou, China
IL-1β	1:500	Cloud-clone crop, Co., Ltd., Wuhan, China
IL-10	1:500	Cloud-clone crop, Co., Ltd., Wuhan, China
p-Nrf2	1:1000	HuaAn Biotechnology Co., Ltd., Hangzhou, China
Nrf2	1:1000	HuaAn Biotechnology Co., Ltd., Hangzhou, China
HO-1	1:500	Boster Biological Technology, Wuhan, China
TRAF6	1:1000	HuaAn Biotechnology Co., Ltd., Hangzhou, China
NF-κB p65	1:500	Abcam, Cambridge, UK
p-ERK	1:1000	Cell signaling technology Inc, Beverly, MA, USA
ERK	1:1000	Cell signaling technology Inc, Beverly, MA, USA
p-JNK	1:1000	Cell signaling technology Inc, Beverly, MA, USA
JNK	1:1000	Cell signaling technology Inc, Beverly, MA, USA
p-p38	1:1000	Cell signaling technology Inc, Beverly, MA, USA
p38	1:1000	Cell signaling technology Inc, Beverly, MA, USA
β-actin	1:10,000	Abclonal, Biotechnology Co., Ltd., Wuhan, China

## Data Availability

Data is contained within the article.
